# Rate of broad-spectrum antibiotic overuse in patients receiving outpatient parenteral antibiotic therapy (OPAT)

**DOI:** 10.1017/ash.2021.204

**Published:** 2021-10-22

**Authors:** Jessa R. Brenon, Stephanie E. Shulder, Sonal S. Munsiff, Colleen M. Burgoyne, Angela K. Nagel, Kelly E. Pillinger

**Affiliations:** 1 Johns Hopkins Specialty Infusion Services, Johns Hopkins Home Care Group, Baltimore, Maryland; 2 Department of Pharmacy, University of Rochester Medical Center, Rochester, New York; 3 Division of Infectious Diseases, Department of Medicine, University of Rochester Medical Center, Rochester, New York; 4 Department of Pharmacy Practice and Administration; Department of Pharmacy, University of Rochester Medical Center, Rochester, New York; 5 Wegmans School of Pharmacy, St John Fisher College, Rochester, New York

## Abstract

Broad-spectrum antibiotics with once-daily dosing are often chosen for outpatient parenteral antibiotic therapy (OPAT) due to convenience even when narrower-spectrum antibiotics are appropriate. At our institution, up to 50% of select broad-spectrum OPAT regimens had potential to be narrowed, highlighting the need to re-evaluate regimens for de-escalation prior to discharge.

Outpatient parenteral antimicrobial therapy (OPAT) is a widely adopted treatment modality.^1–4^ Despite an increased focus on antimicrobial stewardship, data examining the judicious use of antimicrobials in the OPAT setting are limited. To better understand current practice and opportunities for improvement, we sought to identify the percentage of patients within our OPAT program treated with select broad-spectrum antibiotic regimens whose treatment could have been further narrowed based on the culture and susceptibility (C&S) report. We reviewed the documented reason(s) for broad-spectrum therapy when a narrower option was available, and we compared patients who received broad-spectrum therapy when a narrower option was available to those who received narrow-spectrum agents.

## Methods

This retrospective cohort study included adult inpatients aged >18 years who were enrolled in OPAT and were evaluated by the infectious disease (ID) consultation service and discharged between January 1, 2019, and June 30, 2019, from 2 hospitals within the University of Rochester Medical Center. Inpatients with infections due to organisms for which C&S reports were available, and who were on select intravenous antibiotics (ampicillin, ampicillin-sulbactam, cefazolin, ceftriaxone, daptomycin, ertapenem, meropenem, nafcillin, penicillin, piperacillin-tazobactam, and vancomycin) were included. These antibiotics were selected based on a prior data query, which identified that most OPAT patients were prescribed these agents at discharge. Susceptibilities were not required for *Actinomyces, Streptococcus* spp, *Haemophilus* spp, anaerobes or *Corynebacterium* spp, or coagulase-negative *Staphylococcus* spp (if considered a contaminant). Patients on OPAT regimens that included oral antibiotics (not including rifampin or metronidazole) were excluded. The institutional review board approved this study and waived informed consent.

### Classification of antibiotic regimens

National Healthcare Safety Network Antibiotic Use and Resistance antimicrobial groupings were extrapolated to create 4 classes to define antimicrobial de-escalation in this study.^5^ Broad-spectrum class 1 agents (daptomycin and meropenem) were considered the most broad, followed by class 2 agents (ertapenem, piperacillin-tazobactam, and vancomycin), then a class 3 agent (ceftriaxone), with the least broad being the narrow-spectrum class (ampicillin, ampicillin-sulbactam, cefazolin, nafcillin, and penicillin). Regimens with >1 antibiotic were classified according to the broadest-spectrum agent prescribed. Based only on the C&S report, all broad-spectrum regimens were subdivided by 2 independent ID pharmacist reviewers into either the best-available-therapy group (ie, no appropriate narrower-spectrum option) or the group who had broad-spectrum therapy with a narrower available option available. An ID physician adjudicated to resolve any discrepancies. No additional clinical factors, such as antibiotic indication or antibiotic drug allergies, were taken into consideration during the classification of these antibiotic regimens.

### Data analysis

Demographics were summarized with descriptive statistics. The Fisher exact test was used for categorical variables and Mann-Whitney *U* testing was used for continuous variables. Analyses were conducted using R Commander software (Boston, MA).

## Results

In total, 113 patients were included in the study: 64 patients received broad-spectrum antibiotics and 49 patients received narrow-spectrum antibiotics. Of 64 OPAT patients on select broad-spectrum antibiotics, the treatments of 32 patients (50%) had the potential to be further narrowed. Of these 32 patients, the treatments of 30 patients (94%) had the potential to be narrowed to an agent in the narrow-spectrum class. For 2 patients, no narrow-spectrum agent was appropriate based on the C&S reports. Ceftriaxone regimens comprised 24 (75%) of 32 patients in the group who received broad-spectrum therapy with a narrower available option (Table 1). Ceftriaxone regimens comprised 13 (54%) of 24 regimens used to treat monomicrobial *Streptococcus* spp infections. Reasons for selecting a broad-spectrum regimen when a narrower regimen could have been prescribed were undocumented in 71% of cases; however, where documentation was available, convenience (9%) and patient allergies or intolerances (9%) were the most common reasons. No significant difference was found in all-cause 30-day readmission rates between the broad-spectrum therapy with a narrower available option and the narrow-spectrum class groups (19% vs 20%, respectively; *P* = 1.00).

**Table 1.** Subanalysis of Baseline Characteristics for Broad-Spectrum Therapy with a Narrower Available Option Group Versus Narrow-Spectrum Class Group

Characteristic	Patients Who Received Broad-Spectrum Therapy With a Narrower Available Option (n = 32)^a^	Patients Who Received Narrow-Spectrum Class of Therapy (n = 49^a^)	*P* Value^b^
Indications for antibiotics			
Bloodstream infection	7 (19.4)	21 (38.9)	.041
Organisms			
*Staphylococcus aureus*	11 (20.4)	30 (56.6)	<.001
*Streptococcus* spp	15 (27.8)	7 (13.2)	.051
*Enterobacterales*	13 (24.1)	1 (1.9)	<.001
No. of organisms identified			
1	23 (71.9)	45 (91.8)	.019
≥2	9 (28.1)	4 (8.2)	…
Hospital LOS, median (IQR)	11 (6–14)	7 (5–9)	.005
Antibiotics prescribed on discharge			
Cefazolin	0 (0.0)	33 (67.3)	…
Ceftriaxone	24 (75.0)	0 (0.0)	…
Penicillin	0 (0.0)	12 (24.5)	…
Daptomycin	3 (9.4)	0 (0.0)	…
Piperacillin-tazobactam	3 (9.4)	0 (0.0)	…
Ertapenem	2 (6.3)	0 (0.0)	…
Frequency of antibiotic regimens			
Once daily	26 (81.3)	0 (0.0)	<.001
Twice daily	2 (6.3)	1 (2.0)	.3430
TID	0 (0.0)	29 (59.2)	<.001
QID	1 (3.1)	1 (2.0)	.6370
Continuous or extended infusion	2 (6.3)	15 (30.6)	.0070
Given with HD	1 (3.1)	3 (6.1)	.4817

Note. IQR, interquartile range; HD, hemodialysis; LOS, length of stay; TID, 3 times per day; QID, 4 times per day.

a All statistics are expressed as no. (%) unless otherwise stated.

b Statistical significance: *P* ≤ .05.

## Discussion

Half of patients enrolled in our OPAT service on select broad-spectrum regimens had the potential to have their regimen further narrowed. This finding supports current literature suggesting opportunity exists for certain broad-spectrum OPAT regimens to be narrowed, although the magnitude of this issue is not well defined.^6,7^ In a retrospective review, Britt et al^6^ assessed the continuation of broad-spectrum “ease of administration (EOA) regimens” of daptomycin or ertapenem upon hospital readmission during or immediately following OPAT, and subsequently de-escalated EOA regimens in 28% of cases. In a recent Australian antimicrobial prescribing survey audit pilot-tested in OPAT and hospital-in-the-home (HITH) settings, Friedman et al^7^ determined that 11% of 1,154 antibiotic prescriptions were inappropriate and that 9% were unnecessarily broad spectrum. The rationale for the use of such broad-spectrum agents was unknown in many circumstances. Although both studies highlight opportunity for antimicrobial stewardship intervention in OPAT, Britt et al^6^ did not quantify the rate of opportunity beyond just daptomycin and ertapenem use, and Friedman et al^7^ were not able to associate antimicrobial use with the microbiology data that may have influenced prescribing. These factors could explain the higher rate of antimicrobial stewardship de-escalation opportunity in our study. Our study is unique in that it included a wider range of broad-spectrum antibiotics to assess de-escalation opportunities according to microbiology data. In addition, although our data were limited, our findings further highlight the need to identify the reasons why broader-spectrum options are chosen when narrower agents exist.

A subgroup analysis identified that patients in the broad-spectrum therapy with a narrower available option group were more likely to have *Streptococcus* spp, *Enterobacterales,* and polymicrobial infections versus the narrow-spectrum class group. Most of these regimens included ceftriaxone for the treatment of monomicrobial *Streptococcus* spp infections when a penicillin or first-generation cephalosporin could have been used. In addition, a significant number of patients in the narrow-spectrum group were discharged on thrice-daily-dose regimens compared to the once-daily regimens in the broad-spectrum therapy with a narrower available option group. These findings suggest that providers and patients are willing and able to use more frequently dosed narrow-spectrum regimens under certain circumstances.

This study had several limitations. One major limitation, given the retrospective nature of this study, was the absence of reasons documented by providers for selecting a broader-spectrum regimen when a narrower-spectrum option was available, as well as the lack of additional investigation into potential reasons. Patient-specific factors, such as feasibility of certain regimens, may have had an impact on regimen selection that was not accounted for in our study. It would be helpful to conduct a prospective study aimed at collecting this information from the teams in real time, or requiring ID-team documentation of reasons for regimen selection to better understand barriers to prescribing narrow-spectrum antibiotics for OPAT. Additionally, patient allergy information and antibiotic indications were not considered when investigators were deciding whether broad-spectrum regimens could be further narrowed. This aspect of our study may explain the higher rate of patients in the broad-spectrum therapy with a narrower available option group seen in our study in comparison to other studies. Lastly, ID pharmacists made recommendations when able on rounds, but antimicrobial stewardship intervention on OPAT regimen selection was limited.

In conclusion, these findings demonstrate that a potentially large number of patients enrolled in OPAT on select broad-spectrum regimens could have had their regimen further narrowed. Thus, opportunities exist for antimicrobial stewardship review of OPAT regimens prior to discharge to assess barriers and to determine whether de-escalation is possible. Ceftriaxone-containing regimens were identified as the most common regimen with de-escalation potential.

## References

[1] Norris AH , Shrestha NK , Allison GM , et al. 2018 IDSA clinical practice guideline for the management of outpatient parenteral antimicrobial therapy. Clin Infect Dis 2013;68:e1–e35. 10.1093/cid/ciy745 30423035

[2] Winters RW. Home infusion therapy industry: an overview. In: Connors RB , Winters RW , editors. Home Infusion Therapy: Current Status and Future Trends. Chicago: American Hospital Publishing; 1995:1–15.

[3] Tice A. Outpatient parenteral antibiotic therapy (OPAT) in the United States: delivery models and indications for use. Can J Infect Dis Med Microbiol 2000. doi: 10.1155/2000/676915.

[4] Mahoney MV , Ryan KL , Alexander BT. Evaluation of OPAT in the age of antimicrobial stewardship. Curr Treat Options Infect Dis 2020;12:158–177.

[5] Antibiotic use and resistance (AUR) module. Centers for Disease Control and Prevention website. https://www.cdc.gov/nhsn/PDFs/pscManual/11pscAURcurrent.pdf. Published 2019. Accessed October 9, 2019.

[6] Britt RS , Lasalvia MT , Padival S , Patel PV , McCoy C , Mahoney MV. OPAT or No-PAT? Evaluation of outpatient parenteral antimicrobial therapy (OPAT) patients receiving daptomycin or ertapenem for “ease of administration.” Open Forum Infect Dis 2018;5:S553–S554. 10.1093/ofid/ofz496 PMC7047952 32128338

[7] Friedman ND , Lim SM , James R , et al. Measuring antimicrobial prescribing quality in outpatient parenteral antimicrobial therapy (OPAT) services: development and evaluation of a dedicated national antimicrobial prescribing survey. JAC Antimicrob Resist 2020. doi: 10.1093/jacamr/dlaa058. PMC8210186 34223015

